# N‐methyl‐D‐aspartic acid receptor 2A functionalized stationary phase: A reliable method for pursuing potential ligands against Alzheimer's disease from natural products

**DOI:** 10.1111/cns.14101

**Published:** 2023-01-27

**Authors:** Yuan‐Yuan Chen, Yan Xue, Jia‐Tai Yin, Le‐Jing Qu, Hao‐Peng Li, Qian Li, Xin‐Feng Zhao

**Affiliations:** ^1^ College of Life Sciences Northwest University Xi'an China

**Keywords:** affinity chromatography, Alzheimer's disease, drug discovery, natural products, NMDA receptor

## Abstract

**Aims:**

N‐methyl‐D‐aspartic acid (NMDA) receptors play subunit‐specific role in central neuronal development. However, insights into the pharmacological modulation of NMDA receptors were mainly lack of subunit and synaptic selectivity. The purpose of the present study was to develop a novel strategy to rapidly recognize NMDA subunit 2A (NMDA‐2A) ligands from natural products and provide subunit‐selective drug candidates for Alzheimer's disease (AD).

**Methods:**

The recombinant NMDA‐2A containing a tag of epidermal growth factor receptor (EGFR) was expressed in *Escherichia coli* cells and immobilized on ibrutinib‐modified microspheres based on the specific reaction between EGFR and its inhibitor ibrutinib. A novel affinity stationary phase was synthesized to screen NMDA‐2A ligands from *Gardenia jasminoides* Ellis.

**Results:**

The immobilized receptor column exhibited excellent receptor selectivity and ligand‐binding activity. Crocetin was screened by using this method. In a cellular model of AD, the protein level of NMDA‐2A was significantly decreased compared with the control group, while treatment with crocetin significantly increased NMDA‐2A level in a concentration‐dependent manner, confirming that crocetin could bind to NMDA‐2A in vitro.

**Conclusion:**

In the present study, we developed a reliable method for the rapid identification of NMDA‐2A ligands from natural products, which may be used as a platform for new drug discovery to generate high‐quality drug candidates.

## Graphical abstract

(i) The modification of ibrutinib; (ii) the preparation of NMDA‐2A column and its application in bioactive compound identification from *Gardenia jasminoides* Ellis; and (iii) the reaction between ibrutinib‐modified microspheres and EGFR‐tagged NMDA‐2A.

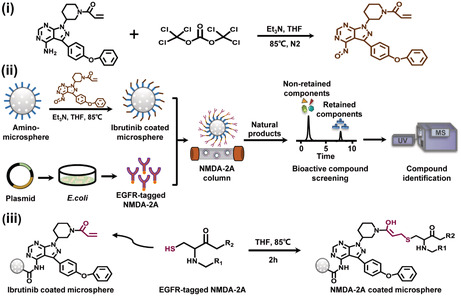

## 1 INTRODUCTION

Alzheimer's disease (AD) is a debilitating and heritable neurological condition with an expectation to blossom into an urgent public health concern in elders.^1^ The worldwide average human life expectancy has increased at a rapid rate with the advances in healthcare, whereas there is a high risk to suffer from AD.^2^, ^3^, ^4^ Despite the profound negative impact of AD on human health, available treatments for AD are effective only in offering short‐term symptomatic relief without addressing the factors responsible for neurodegeneration. Adverse effects of current drugs and their questionable efficiency/cost ratios remain a concern. Moreover, the accumulation of amyloid‐β (Aβ) peptide is a hallmark of AD pathology, while 13 of 15 phase III trials in 2007–2020 targeting Aβ plaque failed to demonstrate clinical efficacy.^5^ The widespread failure of Aβ plaque‐centered therapies triggered a broader perspective on the pathogenesis of AD.

N‐methyl‐D‐aspartic acid (NMDA) receptor subunit 2A (NMDA‐2A) are primarily existing in synapses, whereas extrasynaptic NMDA receptors typically lack subunit 2A and contain mainly subunit 2B.^6^ Although the roles of extrasynaptic and synaptic NMDA receptors in AD are not fully understood, synaptic NMDA‐2A has been reported to exert neuroprotective function; in addition, extrasynaptic NMDA receptors containing subunit 2B is found to mediate neurotoxicity in AD and other brain disorders.^6^ Therefore, NMDA‐2A represented an attractive and alternative target that selectively enhanced synaptic NMDA receptor function while avoiding extrasynaptic neurotoxicity.

*Gardenia jasminoides* Ellis is an evergreen shrub that distributes in the tropical and subtropical areas of the world, the desiccative ripe fruits of which are widely used as not only a natural colorant in food products including confectioneries and noodles, but also a functional health food for making tea and cooking porridge.^7^ In addition, previous studies demonstrated that the fruit of *G. jasminoides* Ellis possessed many beneficial properties and was broadly used for the prevention and treatment of various diseases, such as hypertension,^8^ hepatic injury,^9^ and metabolic disorders.^10^ Notably, *G. jasminoides* Ellis extract was reported to alleviate cognitive deficits in both APP/PS1 transgenic mice^11^ and Aβ‐induced mouse models.^12^ However, the functional components and underlying mechanisms against cognitive decline are still undefined.

In the present study, we expressed NMDA‐2A containing an epidermal growth factor receptor (EGFR) tag in *Escherichia coli* and realized NMDA‐2A immobilization based on covalent interaction of EGFR and its inhibitor ibrutinib. A novel affinity stationary phase was synthesized for NMDA‐2A ligand identification from *G. jasminoides* Ellis. The specificity and reliability of NMDA‐2A column were examined by different methods before its application in screening ligands. Rat pheochromocytoma (PC12) cells incubated with Aβ_25‐35_ served as an AD model to verify whether the screened compound could bind to NMDA‐2A and preliminarily evaluate its effect in vitro.

## 2 METHODS

### 2.1 The preparation of stationary phase containing NMDA‐2A

Amino polystyrene microspheres (pore size 300 Å, amine surface density 450 μmol/g, and particle size 7 μm) were provided by Knowledge & Benefit Sphere Tech. Co., Ltd. NMDA‐2A was immobilized on ibrutinib‐modified microsphere surface through a one‐step immobilization strategy. Briefly, the ibrutinib (303 mg, 1.5 eq) was reacted with triphosgene (202 mg, 1.5 eq) to generate isocyanate at 85°C under a stream of nitrogen gas. After reacting isocyanate with 1 g amino polystyrene microspheres at 85°C for 2 h, ibrutinib was modified on microsphere surface. After washing ibrutinib‐modified microspheres with tetrahydrofuran, petroleum ether, and phosphate‐based buffer pH 7.4, respectively, microspheres were collected for protein immobilization. Based on the highly specific reaction between EGFR‐tag and its inhibitor ibrutinib, NMDA‐2A containing an EGFR‐tag was loaded on microsphere surface by directly mixing ibrutinib‐modified microspheres with the supernatant of cell lysates at room temperature for 1 h. After washing NMDA‐2A‐modified microspheres with phosphate‐based buffer pH 7.4, the microspheres were preserved in 45 mL of phosphate‐based buffer pH 7.4 to maintain protein activity. The NMDA‐2A column was therefore prepared by packing 18 mL of phosphate‐based buffer pH 7.4 containing NMDA‐2A‐modified microspheres into stainless steel columns (4.6 mm × 5.0 cm) under a pressure of 300 bar using a chromatographic column packing machine of ZZXT‐A. The relative content of NMDA‐2A in supernatant of cell lysate was 9.68%, and the total protein concentration of the supernatant determined by bicinchoninic acid assay was 84.40 mg/mL. Therefore, about 8.17 mg/g (6.59 × 10^−8^ mol) of NMDA‐2A protein was attached on the surface of microspheres.

### 2.2 The identification of NMDA‐2A ligands from *Gardenia jasminoides* Ellis extract

*Gardenia jasminoides* Ellis (Rubiaceae) was bought from China Beijing Tongrentang Group Co., Ltd. The extract of *G. jasminoides* Ellis was prepared with reference to the method recorded in Pharmacopeia of the People's Republic of China (2020) with minor modification. Briefly, 10 g of *G. jasminoides* Ellis was broken down to powder by the electric disintegrator, and the powder was soaked with 100 mL of ethanol for 24 h. After sonication treatment for 20 min, the sample solution was condensed to 50 mL (0.2 g/mL) through rotary evaporator. Ammonium acetate buffer (30 mM, pH 7.4) was the mobile phase, and the flow rate was 0.8 mL/min under the detection wavelength of 254 nm during the ligand identification from *G. jasminoides* Ellis extract. The peak that retained on the column was identified via LCMS‐8045 (Shimadzu) through an Inertsil C_18_ column (5 μm, 150 mm × 4.6 mm). Mass spectrum analysis was conducted in negative ion mode over a mass scan range of 50–700 m/z, and the temperature of drying gas was 350°C.

### 2.3 Western blot analysis

Total protein of cells was extracted by radioimmunoprecipitation assay (RIPA) lysis buffer (Cat: R0010) with phenylmethanesulfonyl fluoride (Cat: ST506), and quantified by BCA protein assay kit (Cat: 10136–1). The β‐tubulin antibody (Lot: 334077) was used as the loading control for Western blot analysis to confirm equivalent protein loading in different groups. Same amounts of proteins derived from cell lysates were resolved by SDS‐PAGE and transferred onto nitrocellulose membranes. Nonspecific binding was blocked by 5% skim milk in phosphate buffer saline at room temperature for 2 h. After washing three times with tris‐buffered saline Tween‐20 (TBST), the membranes were incubated with rabbit anti‐NMDA‐2A antibody (Lot: 00083510) diluted 1:800 in TBST and rabbit anti‐tubulin antibody (Lot: 334077) diluted 1:1000 in TBST at 4°C overnight. In addition, the membranes were washed three times with TBST and incubated with corresponding secondary antibodies conjugated with horseradish peroxidase at room temperature for another 2 h. Enhanced chemiluminescence Western blot detection system (GE Healthcare) was utilized to visualize antibody‐binding conjugates. Image‐J software (1.8.0) was used to quantify the bands.

### 2.4 Statistical analysis

All data were expressed as mean ± standard error mean (SEM). Shapiro–Wilk test was used to assess whether data followed a normal (Gaussian) distribution. Comparisons between different groups were analyzed with one‐way ANOVA followed by Tukey's test using GraphPad Prism software (8.0, USA). *p*‐values <0.05 were recognized as statistically significant.

## 3 RESULTS

### 3.1 Expression of EGFR‐tagged NMDA‐2A in *Escherichia coli* cells

**FIGURE 1:**
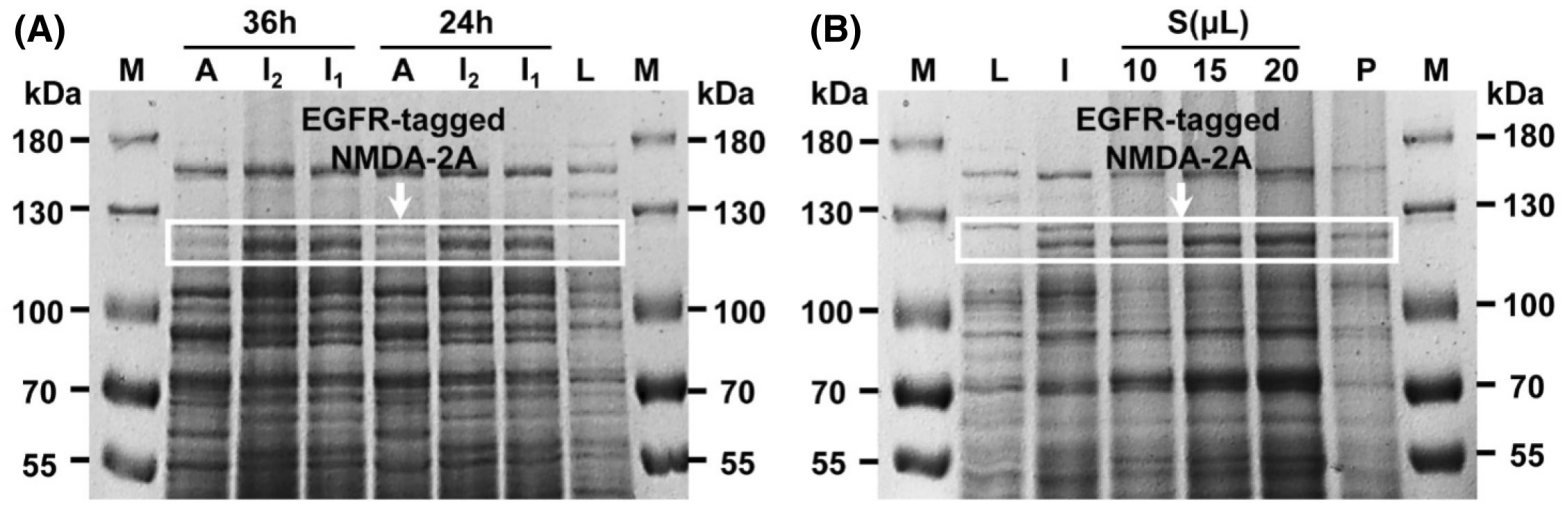
The expression of NMDA‐2A by sodium dodecyl sulfate polyacrylamide gel electrophoresis analysis. (A) The screening of the best expression conditions for NMDA‐2A. Lane M: protein marker, Lane A: auto‐induction medium, Lane I_2_: 1.0 mM IPTG, Lane I_1_: 0.5 mM IPTG, Lane L: Luria‐Bertani medium; (B) The expression of NMDA‐2A at 0.5 mM IPTG/24 h/37°C. Lane M: protein marker, Lane L: Luria‐Bertani medium, Lane I: 0.5 mM IPTG, Lane S: supernatant, Lane P: precipitation.

As illustrated in Figure 1A, a remarkable new sharp band of 124 kDa appeared in LB mediums with 0.5 mM IPTG and 1.0 mM IPTG. However, it was almost undetectable in auto‐induction medium, suggesting that LB medium in the presence of IPTG was more suitable to induce NMDA‐2A expression. In addition, there was insignificant difference between protein level induced by 0.5 mM IPTG and 1.0 mM IPTG, and the level of receptor expression was similar at 24 and 36 h. Therefore, 0.5 mM IPTG/24 h/37°C was selected to induce NMDA‐2A expression. Moreover, the band of 124 kDa was also visible in the supernatant compared with the precipitation, and it became clearer with the increase of sample loading, hinting that the protein was mainly distributed in the supernatant of *Escherichia coli* BL21 (DE3) cell lysates (Figure 1B). Taken together, we successfully expressed EGFR‐tagged NMDA‐2A in LB medium with 0.5 mM IPTG at 37°C for 24 h, and the protein was primarily existed in the supernatant of cell lysates.

### 3.2 Morphology and elemental analysis of stationary phase containing NMDA‐2A

**FIGURE 2:**
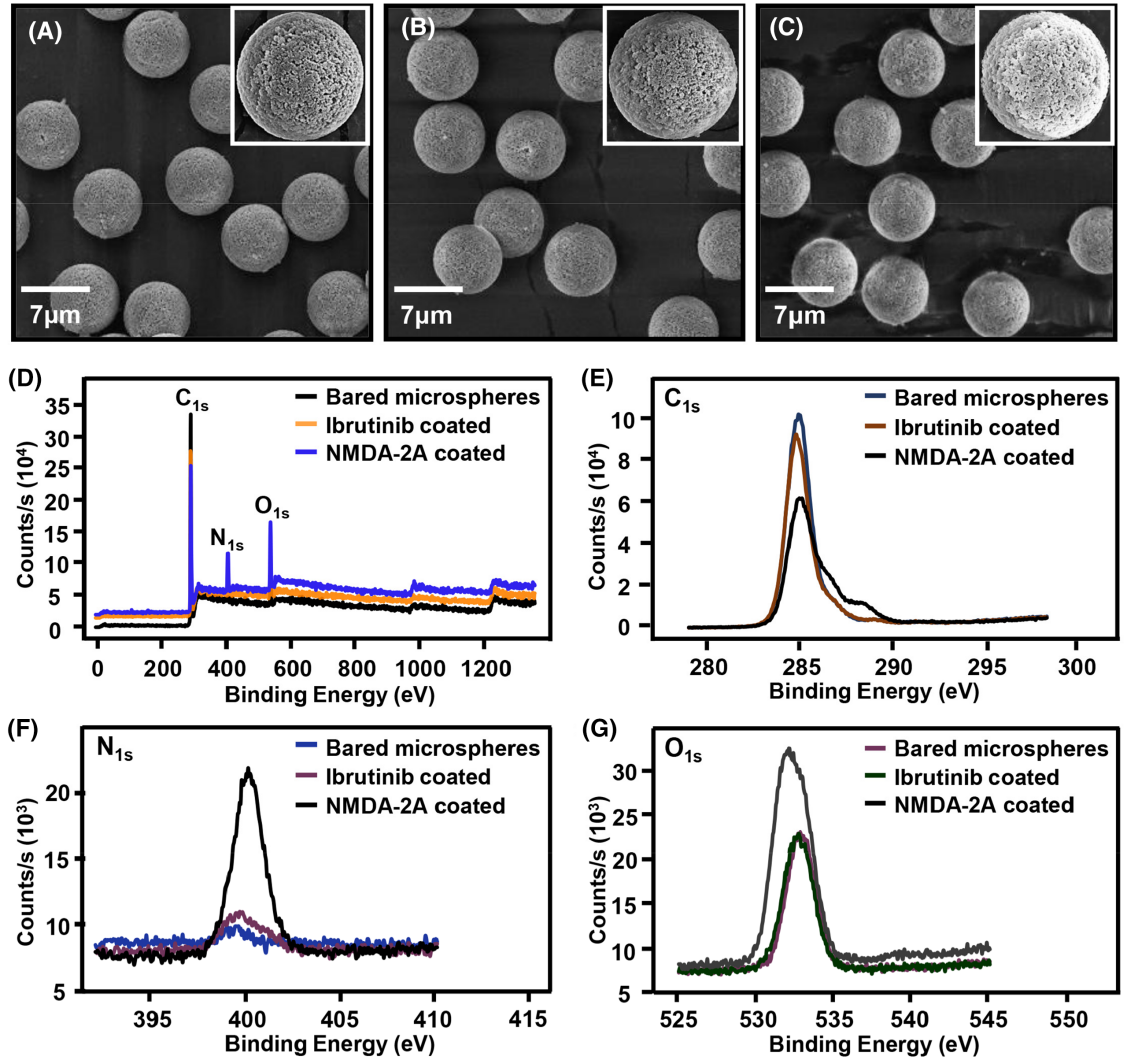
The surface characterization of NMDA‐2A column. The morphology characterization of bared microspheres (A), ibrutinib‐coated microspheres (B) and NMDA‐2A‐coated microspheres (C) by scanning electron microscopy; (D‐G) the surface element characterization of bared microspheres, ibrutinib‐coated microspheres, and NMDA‐2A‐coated microspheres by X‐ray photoelectron spectroscopy.

The surface morphology of the stationary phase was evaluated by scanning electron microscope (SEM). As indicated in Figure 2A,B, the images of microsphere surface showed no remarkable changes after modification of ibrutinib owing to the small molecular weight of ibrutinib. However, the surface of microspheres became rougher when NMDA‐2A was modified, suggesting that NMDA‐2A had been successfully coated on the microsphere surface (Figure 2C). Similar results were observed in immunofluorescence studies. As shown in Figure S1, nearly no fluorescent signal was detected on the surface of bared microspheres and ibrutinib‐modified microspheres. However, obviously brighter fluorescence appeared on the surface of NMDA‐2A‐modified microspheres, confirming the successful modification of NMDA‐2A on microsphere surface.

Moreover, there were four atoms including C, H, N, and O in bared microspheres (amino polystyrene microspheres). We also examined alterations in C, N, and O contents after the modification of ibrutinib and NMDA‐2A by X‐ray photoelectron spectroscopy (XPS) as H atom content cannot be detected by XPS. In the full spectrum (Figure 2D), electron binding energy peaks of C_1s_, N_1s_, and O_1s_ were observed. As shown in Figure 2 E, the binding energy peak of 284.9 eV was dominantly attributed to the C atom in surface aliphatic carbon (C‐C).^13^ The binding energies of 400.68 and 532.28 eV were mainly attributed to the N and O elements in N‐H^14^ and hydroxyl oxygen (C‐O),^15^ respectively. The surface content of C atom was 89.03% in bared microspheres, while its content was gradually decreased in ibrutinib‐modified microspheres (84.38%) and NMDA‐2A‐modified microspheres (71.34%). On the contrary, the content of N atom was 2.74% in bared microspheres, while its content was gradually increased in ibrutinib‐modified microspheres (6.67%) and NMDA‐2A‐modified microspheres (12.50%). Overall, the contents of O atom showed little change in ibrutinib‐modified microspheres (8.95%) compared with bared microspheres (8.23%). However, its contents were significantly increased in NMDA‐2A‐modified microspheres (16.16%) due to the abundance of ‐NH_2_ and ‐COOH groups in NMDA receptor (Figure 2F,G). Collectively, these results indicated that NMDA‐2A had been immobilized on microsphere surface.

### 3.3 Specificity and stability of NMDA‐2A column

**FIGURE 3:**
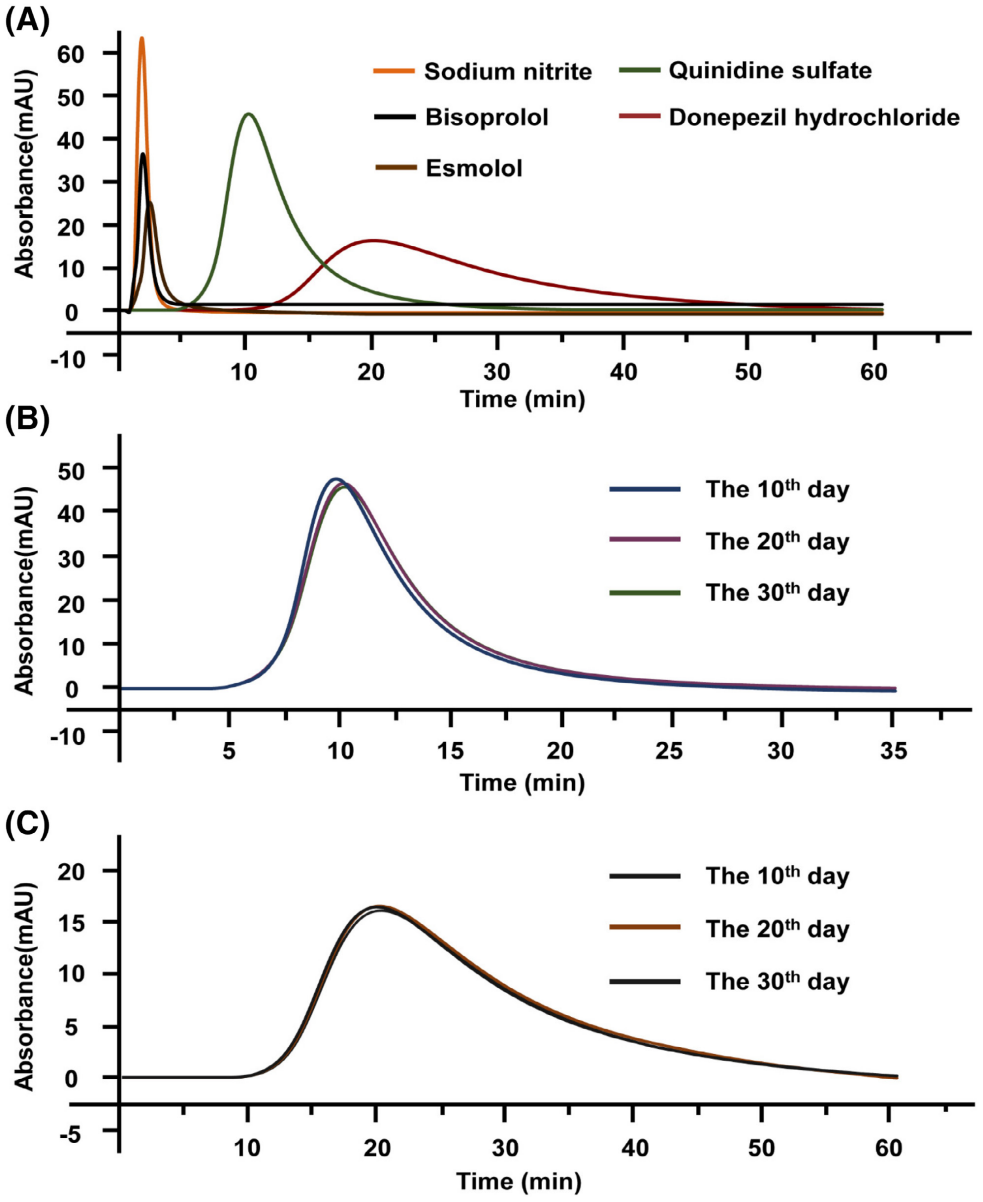
The specificity and stability characterization of NMDA‐2A column. (A) The chromatograms of sodium nitrite, bisoprolol, esmolol, quinidine sulfate, and donepezil hydrochloride on NMDA‐2A column; the chromatograms of quinidine sulfate (B) and donepezil hydrochloride (C) on NMDA‐2A column over thirty days.

Herein, we assessed the specificity of NMDA‐2A column by evaluating the retention time of different ligands under the same mobile phase. As shown in Figure 3A, the system void time was determined to be 1.58 min at the flow rate of 0.4 mL/min by sodium nitrite (with void volume of 0.632 mL), while the retention times of quinidine sulfate and donepezil hydrochloride were much longer with the values of 10.25 and 19.67 min, respectively. Conversely, the retention times of bisoprolol and esmolol (the nonbinding ligands for NMDA receptor) showed little difference from the void time as their retention times were 1.62 and 2.31 min under the same condition. Collectively, these data demonstrated that NMDA‐2A column could recognize its ligands by their retention times with high specificity.

The stability of the affinity stationary phase is also of great importance to the chromatographic efficiency. Taking quinidine sulfate as a probe, we discovered that neither the peak profile nor the retention time was significantly changed with continuous use of 30 days (Figure 3B). Similar observations moved to donepezil hydrochloride, suggesting that NMDA‐2A column was stable in at least 30 days (Figure 3C). Such a long stability in terms of retention times and peak profiles paved the way to apply the NMDA‐2A column in receptor–ligand interaction analysis and screening potential ligands of the receptor with desirable repeatability.

### 3.4 NMDA‐2A–ligand interaction analysis

**FIGURE 4:**
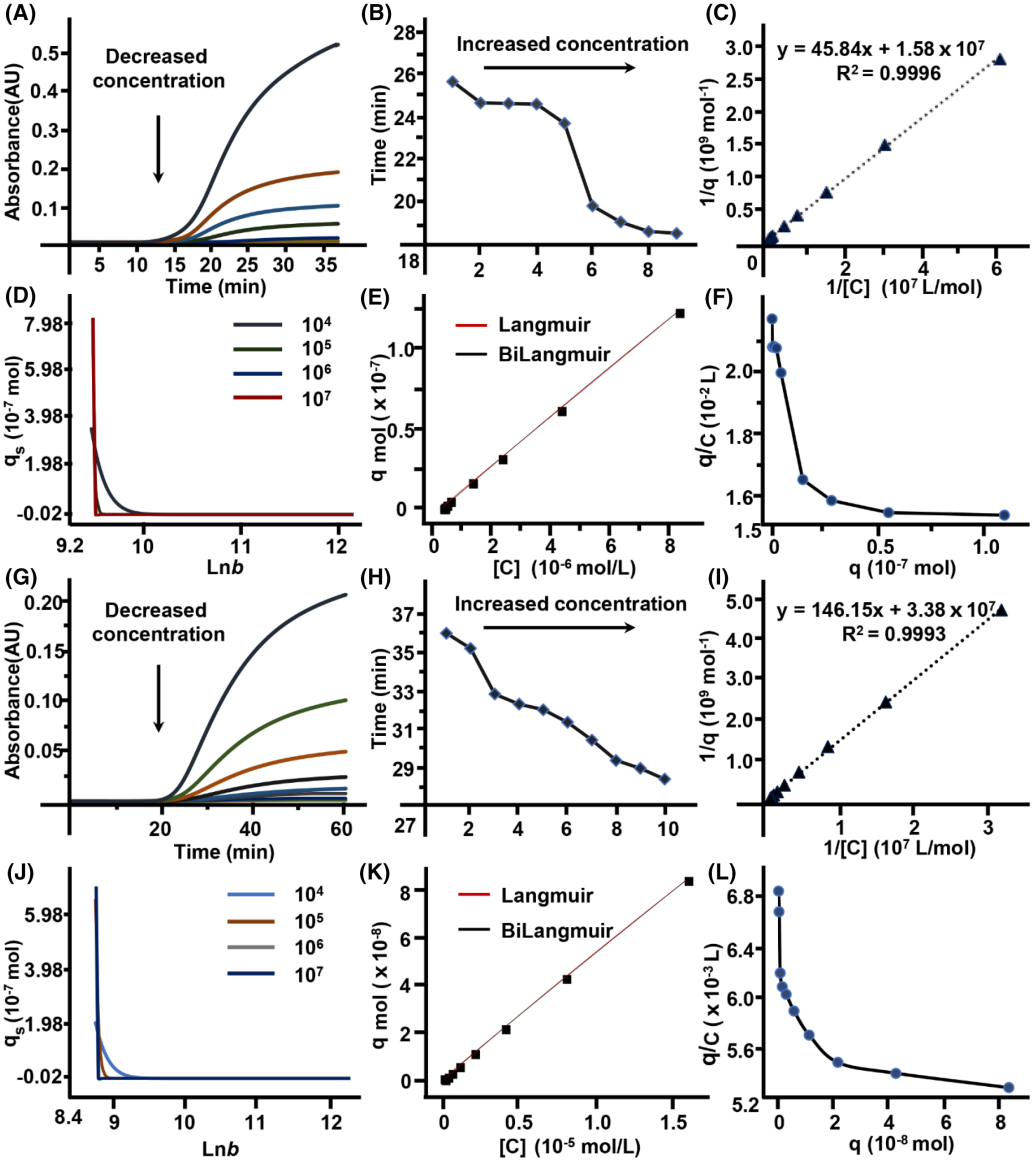
The interaction analysis of quinidine sulfate (15.6 nM, 31.2 nM, 62.5 nM, 125.0 nM, 250.0 nM, 1.0 μM, 2.0 μM, 4.0 μM, and 8.0 μM) with NMDA‐2A and the interaction analysis of donepezil hydrochloride (31.2 nM, 62.5 nM, 125.0 nM, 250.0 nM, 0.5 μM, 1.0 μM, 2.0 μM, 4.0 μM, 8.0 μM, and 16.0 μM) with NMDA‐2A by frontal analysis. The breakthrough curves of quinidine sulfate (A) and donepezil hydrochloride (G) on NMDA‐2A column; the alteration in retention time of quinidine sulfate (B) and donepezil hydrochloride (H) with the increase of ligand concentration; the plots of double‐reciprocal of adsorbed amounts versus concentrations of quinidine sulfate (C) and donepezil hydrochloride (I); the different adsorption isotherms of quinidine sulfate (D–E) and donepezil hydrochloride (J–K); the scatchard plots of quinidine sulfate (F) and donepezil hydrochloride (L) by plotting *q*/[*C*] against *q*. *q* is the adsorbed amount of ligands, and [*C*] represents ligand concentration.

As detailed in Figure 4A,B and Figure 4G,H, the breakthrough time of quinidine sulfate and donepezil hydrochloride gradually decreased with the increase of their concentrations in mobile phase. This indicated that frontal analysis is feasible in realizing the interaction between the two ligands and immobilized NMDA‐2A. As such, we utilized the raw data of frontal analysis to predict their real adsorption models on the column by performing adsorption energy distribution analysis. As illustrated in Figure 4D,J, the peaks became converged and sharp spikes with the increase of iterations from 10^4^ to 10^7^, and there was only one peak for both quinidine sulfate and donepezil hydrochloride. These results demonstrated that the energy distributions of the two ligands were uniform, and their bindings to the column were in line with one‐site model like Langmuir adsorption. To confirm such prediction, we fitted the adsorption data of the two ligands by Langmuir and Bi‐Langmuir models and observed a total overlap between the two fittings (Figure 4 E,K). Thus, we proved that there was only one type of sites for quinidine sulfate and donepezil hydrochloride binding to the receptor. Further regression demonstrated a linear relationship of *y* = 45.84*x* + 1.58 × 10^7^ (*R* ^2^ = 0.9996) by the double‐reciprocal of adsorbed amounts (*q*) versus quinidine sulfate concentrations ([*C*]), by which the association constant was calculated to be (0.34 ± 0.02) × 10^6^ M^−1^ (Figure 4C). Likewise, a linear relationship of *y* = 146.15*x* + 3.38 × 10^7^ (*R* ^2^ = 0.9993) was regressed by the double‐reciprocal of adsorbed amounts (*q*) versus donepezil hydrochloride concentrations ([*C*]), and the association constant was (0.23 ± 0.06) × 10^6^ M^−1^ (Figure 4I). Beyond these observations, the Scatchard plot of quinidine sulfate/donepezil hydrochloride appeared to be biphasic at high and low fractional saturation, suggesting that allosteric modulation of the NMDA‐2A conformations were likely involved in the bindings of the two ligands to the receptor (Figure 4F,L).

**FIGURE 5:**
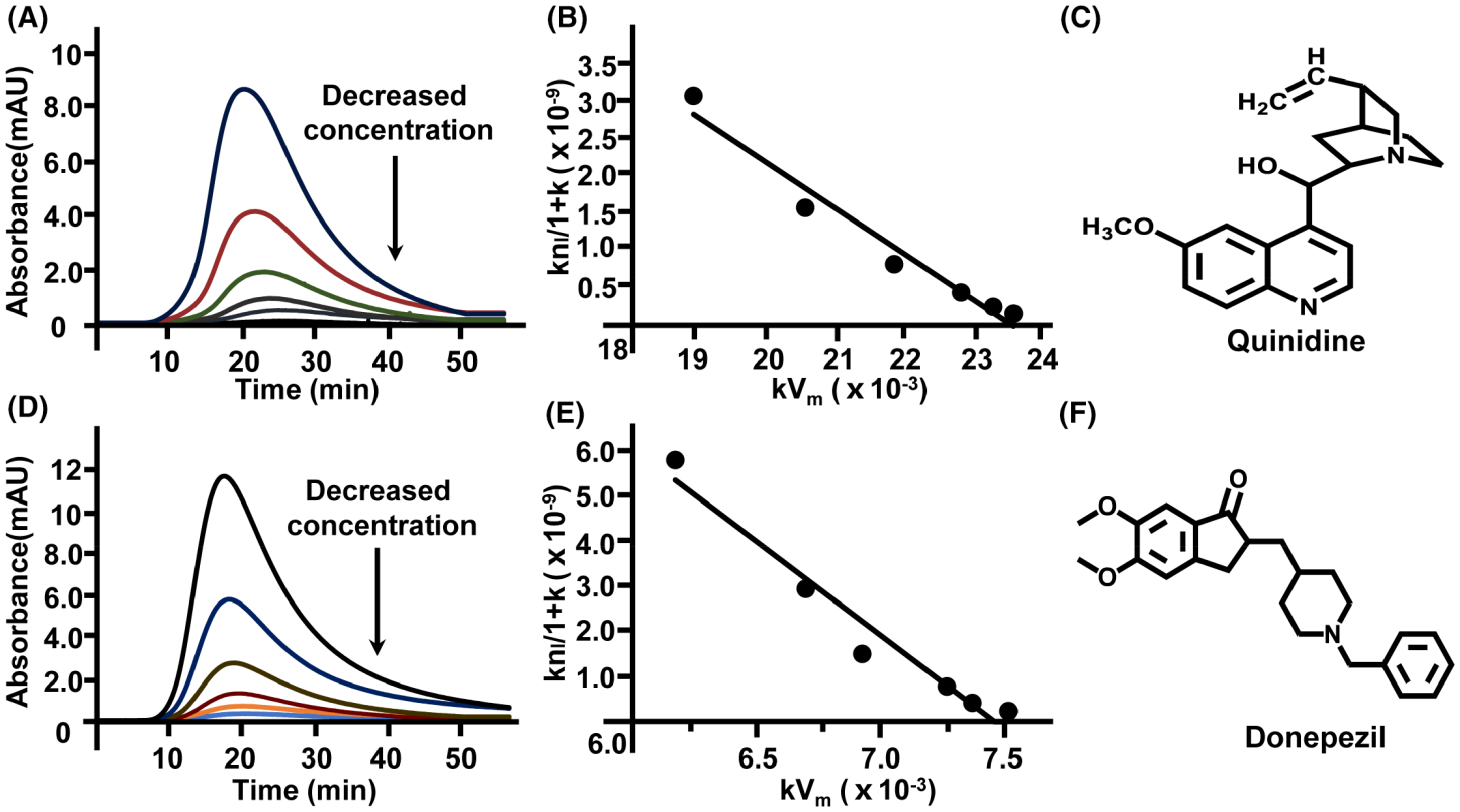
The interactions of quinidine sulfate (10, 20, 40, 80, 160, and 320 μM) and donepezil hydrochloride (20, 40, 80, 160, 320, and 640 μM) with NMDA‐2A by injection amount‐dependent analysis. The chromatograms of quinidine sulfate (A) and donepezil hydrochloride (D) on NMDA‐2A column; the regression curves of quinidine sulfate (B) and donepezil hydrochloride (E) by plotting *k*n_I_/(1 + *k*) versus *k*V_m_; the structure of quinidine sulfate (C) and donepezil hydrochloride (F). *k* is retention factor of the ligand, *n*_*I*_ is the amount of injection solute, and *V*_*m*_ is void volume of chromatographic system.

Except for frontal analysis, we also explored the interaction of NMDA‐2A and its ligand by injection amount‐dependent method. As shown in Figure 5, the retention time of quinidine sulfate and donepezil hydrochloride was gradually decreased with the increasing amounts of the injections, and a linear relationship was observed for each of the two ligands via fitting plots of *k*n_I_/(1 + *k*) to *kV*_*m*_. These data indicated that one type of affinity sites was involved in their bindings to the receptor, which is consistent with those of frontal analysis. The association constant of quinidine sulfate was (1.63 ± 0.14) × 10^6^ M^−1^ with the correlation coefficient (*r* ^2^) of 0.9653. Similarly, the association constant of donepezil hydrochloride was (0.24 ± 0.07) × 10^6^ M^−1^ with the correlation coefficient (*r* ^2^) of 0.9567. Although the binding constants of quinidine sulfate and donepezil hydrochloride obtained from injection amount‐dependent method were different from those of frontal analysis, same affinity ranking was observed (quinidine sulfate > donepezil hydrochloride). Through the literature review, no binding affinities of quinidine and donepezil on NMDA‐2A have been reported. Other methods such as nonlinear chromatography and radioligand‐binding assay will be conducted to confirm our results and evaluate their abilities in calculating the binding parameters of receptor–ligand interactions in the future. Overall, frontal analysis is classical chromatographic method for receptor–ligand interaction analysis with advantages of high accuracy and precision that was first developed by Kasai and Ishii.^16^ However, frontal analysis requires large amounts of drugs to saturate the column, and the analysis time is long. These disadvantages limit its use in ligands that are expensive or hard to obtain. Injection amount‐dependent method was a new methodology for exploring receptor–ligand interactions proposed by our group in 2014 to address the problems of time‐ and drug‐consuming in frontal analysis.^17^ Compared with frontal analysis, injection amount‐dependent method is time‐ and drug‐saving since it was carried out without the saturation of NMDA‐2A column by ligands. In addition, the analysis speed of injection amount‐dependent method is fast, while its accuracy is lower than frontal analysis. Therefore, injection amount‐dependent method is particularly suited for drugs that are expensive or hard to obtain.

### 3.5 Screening NMDA‐2A ligands from *Gardenia jasminoides* Ellis

A mixed sample of T0070907, bisoprolol, crocin I, and ambrisentan (the nonbinding ligands for NMDA receptor) was used to examine the reliability of NMDA‐2A column before NMDA‐2A ligand identification. As indicated in Figure S2, the retention time of the mixed sample was 1.32 min at 230 nm with a flow rate of 0.8 mL/min, showing little difference from the void time (1.18 min) determined by sodium nitrite. However, an obvious retained peak appeared when huperzine A (a ligand of NMDA) was added into the mixed sample of T0070907, bisoprolol, crocin I, and ambrisentan, suggesting that our method was reliable in screening NMDA‐2A ligands.

**FIGURE 6:**
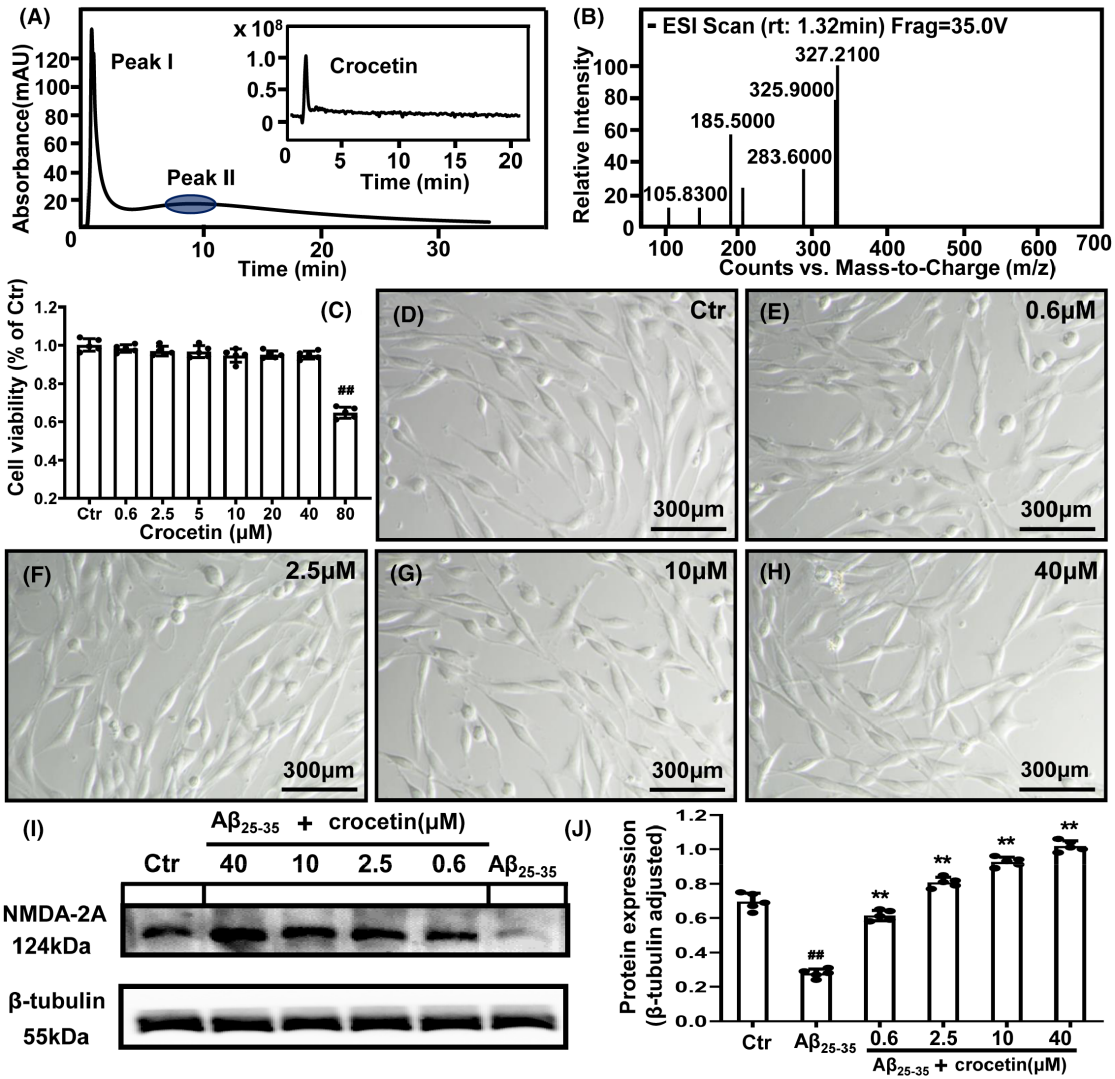
The screening of bioactive compounds from *Gardenia jasminoides* Ellis by NMDA‐2A column and the effect of the screened compound on the protein expression level of NMDA‐2A in rat pheochromocytoma (PC12) cells. (A) The chromatograms of *Gardenia jasminoides* Ellis crude extracts on NMDA‐2A column and total ion current of peak II; (B) the identification of peak II by HPLC‐MS; (C) effects of crocetin on cell viability by MTT assay; (D‐H) the cell morphology in the control cells and different doses of crocetin treated cells; (I‐J) Western blot analysis of NMDA‐2A in different groups. ## = *p* < 0.01 versus the control group; ** = *p* < 0.01 versus the AD group.

The peak II at 8.21 min was recognized as the retained fraction targeting NMDA‐2A (Figure 6A), while peak I at 1.25 min was composed of the nonbinding compounds since its retention time was very close to the chromatographic void time (1.18 min). In order to improve the accuracy of our method, the tip of peak II that highlighted in blue circle instead of the whole peak was collected for bioactive compound identification to avoid the false‐positive results. According to high‐performance liquid chromatography‐mass spectrometry, we found there was only one compound in the tip of peak II. By referring to the standard mass spectra of crocetin, we found this compound was crocetin (m/z 327.2100 [M‐H]^−^) with the retention time of 1.32 min (Figure 6B). According to the literature, there were 17 natural antioxidants from the extract of *Gardenia* fruits (fruit of *G. jasminoides* Ellis). Among them, crocetin, crocin I, and crocin II were the three major components of these 17 compounds.^18^ However, crocin has no effect on both the mRNA gene expression and protein levels of NMDA.^19^ Therefore, we merely screened crocetin from *G. jasminoides* Ellis using NMDA‐2A column.

To further verify whether crocetin could alleviate AD by binding to NMDA‐2A, we carried out Western blot analysis. The model of AD was established by treating PC12 cells with Aβ_25‐35_ for 24 h. The cytotoxicity of crocetin was determined by MTT assay. As shown in Figure 6C, there was no significant difference in cell viability among cells co‐cultured with crocetin at 0.6, 2.5, 5, 10, 20, and 40 μM for 24 h. However, cell viability was significantly decreased in the presence of 80 μM crocetin. Moreover, crocetin at a range of 0.6–40 μM did not induce a significant change of cell morphology during 24‐h incubation (Figure 6D‐H). Therefore, the concentrations at 0.6, 2.5, and 10, 40 μM was chosen for further study. In the in vitro model of AD, the protein level of NMDA‐2A was significantly decreased compared with the control group, while crocetin could increase the protein level of NMDA‐2A in a concentration‐dependent manner (Figure 6I,J), suggesting that crocetin could bind to NMDA‐2A and our method is feasible in screening bioactive compounds with NMDA‐2A activity from natural products.

## 4 DISCUSSION

Alzheimer's disease is a devastating neurodegenerative disease characterized by intracellular neurofibrillary tau tangles and extracellular Aβ plaque accumulation, which is recognized as the largest unmet health problem leading to brain shrinkage.^20^ The available drugs for AD, primarily including acetylcholinesterase inhibitors and memantine, the NMDA receptor antagonist, are only temporarily effective in ameliorating cognitive decline but unable to halt or reverse disease progression.^21^ Therefore, new drugs that could retard or slow AD progression are pressingly needed.

N‐methyl‐D‐aspartic acid receptor plays a central role in memory formation and brain development.^22^ The activation of synaptic NMDA receptors triggers intracellular cascades and facilitates neuronal cell survival. However, excessive extrasynaptic NMDA receptor activation causes excitotoxicity and behavioral deficits through mitochondrial dysfunction, which therefore contributes to AD pathology.^23^ NMDA‐2A is mainly locating in synapses, the activation of which promotes neuronal survival and exerts a neuroprotective effect against neuronal damage.^24^, ^25^, ^26^ Previous studies reported that protein expression of NMDA‐2A was decreased in the hippocampus of rats showing cognitive deficits and AD‐like neuropathological impairments.^27^ Hanson et al.^6^ demonstrated the potential therapeutic benefit of NMDA‐2A enhancement in AD intervention in 2020. However, the study of NMDA receptors is coming mostly from the pharmacological modulation of NMDA receptors with limited subunit and synaptic selectivity, mainly using NMDA receptor antagonists like memantine or MK‐801. As such, progress in strategies aimed at targeting NMDA‐2A may lead to a paradigm shift in new drug discovery for AD treatment.

In the present study, we developed a reliable method for the rapid identification of NMDA‐2A ligands from natural products and screened NMDA‐2A ligands from *G. jasminoides* Ellis using NMDA‐2A column. Wainer et al. have established several affinity stationary phases containing immobilized transporters, transmembrane receptors, or enzymes by physical adsorption over the last decades, while their specificity and long‐term stability need to be improved.^28^, ^29^, ^30^ Herein, we synthesized the affinity stationary phase based on a highly specific covalent reaction between EGFR‐tag and its inhibitor ibrutinib. It was more stable and active than those affinity stationary phases prepared by physical adsorption. Using this NMDA‐2A column, crocetin was screened from *G. jasminoides* Ellis. Previous studies reported that crocetin was a main active constituents of *G. jasminoides* Ellis that possessed multiple pharmacological characteristics, which showed huge potential in combating AD.^31^ For example, crocetin decreased Aβ accumulation in APPsw transgenic mice^32^ and significantly suppressed key molecular pathways of AD pathogenesis concerning toxic Aβ peptide production and Tau misprocessing in two AD neuronal cell culture models.^33^ In addition, crocetin could inhibit Aβ aggregation by stabilizing Aβ oligomers^34^ and accelerate the degradation of Aβ in monocytes from patients with AD.^35^ In the present study, we discovered that crocetin increased the decreased protein level of NMDA‐2A in an in vitro model of AD, suggesting that crocetin may also ameliorate AD by mediating NMDA‐2A, which is consistent with previous studies that crocetin exhibited considerable NMDA receptor affinity.^36^, ^37^

Undoubtedly, natural products are precious sources for new drug discovery.^38^ Actually, about half of all approved new drugs around 1940 to the end of 2014 were coming from natural products or their derivatives.^39^ However, the traditional strategies for drug development from natural sources faced with hurdles like difficulties in isolating pure phytoconstituents and the minute quantities of isolated compounds that were insufficient for subsequent studies. The identification of bioactive compound remains an extremely laborious and time‐consuming process. An average of ten years upward and more than 800 million dollars were taken during the process of drug discovery.^40^ Much time and money were spent on numerous lead compound identification that was discarded during drug discovery. About one in 5000 lead compounds can be advanced through clinical trials and successfully be approved for use.^41^ In addition, some minor or unstable components might be lost during the processes of separation, and the underlying mechanism of the isolated compound is largely unknown, severely retarding their clinical use. Herein, we developed a novel strategy to rapidly screen bioactive compounds with NMDA‐2A activity from natural products, providing subunit‐selective drug candidates for AD. Compared with traditional screening methods, the current method could capture lead compounds from complex system with high chromatographic speed, and their target was known, which may facilitate the development of new drugs. Of note, although we established a potential high‐throughput screening method for ligand identification by collecting the tips of retention peaks with rapid speed, some compounds with NMDA‐2A activity might be neglected since many active components could exist in whole peaks. This may be a disadvantage of our method. A combination of other screening approaches instead of a single method may help to comprehensively explore the chemical composition of *G. jasminoides* Ellis. Moreover, functional assays and in vivo studies of the screened compound remain indispensable to provide reliable data. Given the fact that a natural product cannot be approved as a new drug until proper evidence, there is a great impetus to carry out functional assays and pharmacological studies of crocetin in the future.

## 5 CONCLUSION

In the present study, we synthesized a novel affinity stationary phase by immobilizing NMDA‐2A on ibrutinib‐modified microsphere surface and carried out bioactive compound identification from *Gardenia jasminoides* Ellis using NMDA‐2A column. A new method for rapidly recognizing NMDA‐2A ligands from natural products with high specificity and accuracy was developed by our group, which may offer a far more productive route to screen subunit‐selective drug candidates for AD intervention.

## AUTHOR CONTRIBUTIONS.

Xin‐Feng Zhao conceived and designed this manuscript. Qian Li supervised the project and revised the manuscript. Yuan‐Yuan Chen conducted the experiments and prepared the manuscript. Yan‐Xue gave support in cell culture. Le‐Jing Qu, Jia‐Tai Yin, and Hao‐Peng Li help to edit the manuscript. All authors have read and approved the final manuscript.

## FUNDING STATEMENT

We acknowledged the National Natural Science Foundation of China (21,974,107 and 22,074,118), the Shaanxi Provincial Department of Science and Technology (2020ZDLSF05‐07 and 2022KJXX‐70), and Shaanxi Administration of Traditional Chinese Medicine (2021‐04‐22‐005 and 2022‐SLRH‐YQ‐007) for the financial support of this work.

## CONFLICT OF INTEREST STATEMENT

The authors declare that there is no conflict of interest.

## Supporting information


AppendixS1

## Data Availability

Not applicable.

## References

[1] Scheltens P , de Strooper B , Kivipelto M , Holstege H , Chételat G , Teunissen CE . Alzheimer's disease. Lancet. 2021;397(10284):1577‐1590. 33667416 10.1016/S0140-6736(20)32205-4 PMC8354300

[2] Gupta VB , Chitranshi N , den Haan J , et al. Retinal changes in Alzheimer's disease‐integrated prospects of imaging, functional and molecular advances. Prog Retin Eye Res. 2021;82:100899. 32890742 10.1016/j.preteyeres.2020.100899

[3] Chen YY , Wang MC , Wang YN , et al. Redox signaling and Alzheimer's disease: from pathomechanism insights to biomarker discovery and therapy strategy. Biomark Res. 2020;8:42. 32944245 10.1186/s40364-020-00218-z PMC7488504

[4] Nakamura A , Kaneko N , Villemagne VL , et al. High performance plasma amyloid‐β biomarkers for Alzheimer's disease. Nature. 2018;554(7691):249‐254. 29420472 10.1038/nature25456

[5] Karran E , De Strooper B . The amyloid hypothesis in Alzheimer disease: new insights from new therapeutics. Nat Rev Drug Discov. 2022;21(4):306‐318. 35177833 10.1038/s41573-022-00391-w

[6] Hanson JE , Ma K , Elstrott J , et al. GluN2A NMDA receptor enhancement improves brain oscillations, synchrony, and cognitive functions in dravet syndrome and Alzheimer's disease models. Cell Rep. 2020;30(2):381‐396.e4. 31940483 10.1016/j.celrep.2019.12.030 PMC7017907

[7] Han Y , Wen J , Zhou T , Fan G . Chemical fingerprinting of Gardenia jasminoides Ellis by HPLC‐DAD‐ESI‐MS combined with chemometrics methods. Food Chem. 2015;188:648‐657. 26041243 10.1016/j.foodchem.2015.05.039

[8] Higashino S , Sasaki Y , Giddings JC , et al. Crocetin, a carotenoid from Gardenia jasminoides Ellis, protects against hypertension and cerebral thrombogenesis in stroke‐prone spontaneously hypertensive rats. Phytother Res. 2014;28(9):1315‐1319. 24550159 10.1002/ptr.5130

[9] Fang S , Wang T , Li Y , et al. Gardenia jasminoides Ellis polysaccharide ameliorates cholestatic liver injury by alleviating gut microbiota dysbiosis and inhibiting the TLR4/NF‐κB signaling pathway. Int J Biol Macromol. 2022;205:23‐36. 35176320 10.1016/j.ijbiomac.2022.02.056

[10] Zhong H , Chen K , Feng M , et al. Genipin alleviates high‐fat diet‐induced hyperlipidemia and hepatic lipid accumulation in mice via miR‐142a‐5p/SREBP‐1c axis. FEBS J. 2018;285(3):501‐517. 29197188 10.1111/febs.14349

[11] Zang C , Liu H , Shang J , et al. Gardenia jasminoides J.Ellis extract GJ‐4 alleviated cognitive deficits of APP/PS1 transgenic mice. Phytomedicine. 2021;93:153780. 34607163 10.1016/j.phymed.2021.153780

[12] Zang CX , Bao XQ , Li L , et al. The protective effects of Gardenia jasminoides (fructus gardenia) on amyloid‐β‐induced mouse cognitive impairment and neurotoxicity. Am J Chin Med. 2018;46(2):389‐405. 29433392 10.1142/S0192415X18500192

[13] Hu Y , Dan W , Xiong S , et al. Development of collagen/polydopamine complexed matrix as mechanically enhanced and highly biocompatible semi‐natural tissue engineering scaffold. Acta Biomater. 2017;47:135‐148. 27744068 10.1016/j.actbio.2016.10.017

[14] Du H , Gao X , Ma Q , Yang X , Zhao TS . Cu/PCN metal‐semiconductor heterojunction by thermal reduction for photoreaction of CO2‐aerated H2O to CH3OH and C2H5OH. ACS Omega. 2022;7(19):16817‐16826. 35601319 10.1021/acsomega.2c01827 PMC9118400

[15] Liu Z , Feng X , Wang X , Yang S , Mao J , Gong S . Quercetin as an auxiliary endodontic irrigant for root canal treatment: anti‐biofilm and dentin collagen‐stabilizing effects in vitro. Materials (Basel). 2021;14(5):1178. 33802293 10.3390/ma14051178 PMC7959140

[16] Kasai K , Ishii S . Quantitative analysis of affinity chromatography of trypsin. A new technique for investigation of protein‐ligand interaction. J Biochem. 1975;77(1?):261‐264. 805787

[17] Zhao X , Li Q , Xiao C , et al. Oriented immobilisation of histidine‐tagged protein and its application in exploring interactions between ligands and proteins. Anal Bioanal Chem. 2014;406(12):2975‐2985. 24633512 10.1007/s00216-014-7723-x

[18] Zhuang GD , Gu WT , Xu SH , et al. Rapid screening of antioxidant from natural products by AAPH‐incubating HPLC‐DAD‐HR MS/MS method: a case study of Gardenia jasminoides fruit. Food Chem. 2023;401:134091. 36116299 10.1016/j.foodchem.2022.134091

[19] Adabizadeh M , Mehri S , Rajabpour M , Abnous K , Rashedinia M , Hosseinzadeh H . The effects of crocin on spatial memory impairment induced by hyoscine: role of NMDA, AMPA, ERK, and CaMKII proteins in rat hippocampus. Iran J Basic Med Sci. 2019;22(6):601‐609. 31231486 10.22038/ijbms.2019.30138.7266 PMC6570751

[20] Khandelwal M , Manglani K , Upadhyay P , Azad M , Gupta S . AdipoRon induces AMPK activation and ameliorates Alzheimer's like pathologies and associated cognitive impairment in APP/PS1 mice. Neurobiol Dis. 2022;174:105876. 36162737 10.1016/j.nbd.2022.105876

[21] Scarpini E , Scheltens P , Feldman H . Treatment of Alzheimer's disease: current status and new perspectives. Lancet Neurol. 2003;2(9):539‐547. 12941576 10.1016/s1474-4422(03)00502-7

[22] Jalali‐Yazdi F , Chowdhury S , Yoshioka C , Gouaux E . Mechanisms for zinc and proton inhibition of the GluN1/GluN2A NMDA receptor. Cell. 2018;175(6):1520‐1532.e15. 30500536 10.1016/j.cell.2018.10.043 PMC6333211

[23] Hardingham GE , Bading H . Synaptic versus extrasynaptic NMDA receptor signalling: implications for neurodegenerative disorders. Nat Rev Neurosci. 2010;11(10):682‐696. 20842175 10.1038/nrn2911 PMC2948541

[24] McQuail JA , Beas BS , Kelly KB , et al. NR2A‐containing NMDARs in the prefrontal cortex are required for working memory and associated with age‐related cognitive decline. J Neurosci. 2016;36(50):12537‐12548. 27807032 10.1523/JNEUROSCI.2332-16.2016 PMC5157101

[25] Babaei P . NMDA and AMPA receptors dysregulation in Alzheimer's disease. Eur J Pharmacol. 2021;908:174310. 34265291 10.1016/j.ejphar.2021.174310

[26] Liu Y , Wong TP , Aarts M , et al. NMDA receptor subunits have differential roles in mediating excitotoxic neuronal death both in vitro and in vivo. J Neurosci. 2007;27(11):2846‐2857. 17360906 10.1523/JNEUROSCI.0116-07.2007 PMC6672582

[27] Jin L , Li YP , Feng Q , et al. Cognitive deficits and Alzheimer‐like neuropathological impairments during adolescence in a rat model of type 2 diabetes mellitus. Neural Regen Res. 2018;13(11):1995‐2004. 30233075 10.4103/1673-5374.239448 PMC6183048

[28] Moaddel R , Wainer IW . Immobilized nicotinic receptor stationary phases: going with the flow in high‐throughput screening and pharmacological studies. J Pharm Biomed Anal. 2003;30(6):1715‐1724. 12485712 10.1016/s0731-7085(02)00513-7

[29] Calleri E , Ceruti S , Cristalli G , et al. Frontal affinity chromatography‐mass spectrometry useful for characterization of new ligands for GPR17 receptor. J Med Chem. 2010;53(9):3489‐3501. 20394377 10.1021/jm901691y PMC6201305

[30] Moaddel R , Rosenberg A , Spelman K , et al. Development and characterization of immobilized cannabinoid receptor (CB1/CB2) open tubular column for on‐line screening. Anal Biochem. 2011;412(1):85‐91. 21215722 10.1016/j.ab.2010.12.034 PMC3053438

[31] Guo ZL , Li MX , Li XL , et al. Crocetin: a systematic review. Front Pharmacol. 2022;12:745683. 35095483 10.3389/fphar.2021.745683 PMC8795768

[32] Zhang J , Wang Y , Dong X , Liu J . Crocetin attenuates inflammation and amyloid‐β accumulation in APPsw transgenic mice. Immun Ageing. 2018;15:24. 30450117 10.1186/s12979-018-0132-9 PMC6208089

[33] Chalatsa I , Arvanitis DA , Koulakiotis NS , et al. The crocus sativus compounds trans‐crocin 4 and trans‐crocetin modulate the amyloidogenic pathway and tau misprocessing in Alzheimer disease neuronal cell culture models. Front Neurosci. 2019;13:249. 30971876 10.3389/fnins.2019.00249 PMC6443833

[34] Ahn JH , Hu Y , Hernandez M , Kim JR . Crocetin inhibits β‐amyloid fibrillization and stabilizes β‐amyloid oligomers. Biochem Biophys Res Commun. 2011;414(1):79‐83. 21945434 10.1016/j.bbrc.2011.09.025

[35] Tiribuzi R , Crispoltoni L , Chiurchiù V , Casella A , Montecchiani C , del Pino A . Trans‐crocetin improves amyloid‐β degradation in monocytes from Alzheimer's disease patients. J Neurol Sci. 2017;372:408‐412. 27865556 10.1016/j.jns.2016.11.004

[36] Lechtenberg M , Schepmann D , Niehues M , Hellenbrand N , Wunsch B , Hensel A . Quality and functionality of saffron: quality control, species assortment and affinity of extract and isolated saffron compounds to NMDA and σ1 (σ‐1) receptors. Planta Med. 2008;74(7):764‐772. 18496783 10.1055/s-2008-1074535

[37] Lautenschlager M , Sendker J , Huwel S , et al. Intestinal formation of trans‐crocetin from saffron extract (Crocus sativus L.) and in vitro permeation through intestinal and blood brain barrier. Phytomedicine. 2015;22(1):36‐44. 25636868 10.1016/j.phymed.2014.10.009

[38] Atanasov AG , Zotchev SB , Dirsch VM , International Natural Product Sciences T , Supuran CT . Natural products in drug discovery: advances and opportunities. Nat Rev Drug Discov. 2021;20(3):200‐216. 33510482 10.1038/s41573-020-00114-z PMC7841765

[39] Newman DJ , Cragg GM . Natural products as sources of new drugs from 1981 to 2014. J Nat Prod. 2016;79(3):629‐661. 26852623 10.1021/acs.jnatprod.5b01055

[40] Reichert JM . Trends in development and approval times for new therapeutics in the United States. Nat Rev Drug Discov. 2003;2(9):695‐702. 12951576 10.1038/nrd1178

[41] Balunas MJ , Kinghorn AD . Drug discovery from medicinal plants. Life Sci. 2005;78(5):431‐441. 16198377 10.1016/j.lfs.2005.09.012

