# Supplementary material for: N‐methyl‐D‐aspartic acid receptor 2A functionalized stationary phase: A reliable method for pursuing potential ligands against Alzheimer's disease from natural products
Source: CNS Neurosci Ther. 2023 Jan 27;29(5):1290–9. doi: 10.1111/cns.14101 (PMC10068460; doi:10.1111/cns.14101)
Supplement: Supplementary file 1 — AppendixS1 [file CNS-29-1290-s001.doc]

**N-methyl-D-aspartic acid receptor 2A functionalized stationary phase: a reliable method for pursuing potential ligands against Alzheimer’s disease from natural products**

Running title: A new method for drug candidate discovery

Yuan-Yuan Chen^a^, Yan Xue^a^, Jia-Tai Yin^a^, Le-Jing Qu^a^, Hao-Peng Li^a^, Qian Li^a^, Xin-Feng Zhao^a^,*

^a^ College of Life Sciences, Northwest University, Xi’an 710069, China

*Corresponding author:

Xin-Feng Zhao Professor, PhD, MD

College of Life Sciences, Northwest University

No. 229 Taibai North Road, Xi’an Shaanxi 710069, China

Tel.: 86-29-88303572

Email: zhaoxf@nwu.edu.cn

**Supplemental Methods**

**The expression of NMDA-2A containing an EGFR-tag in *Escherichia coli***

*Escherichia coli* BL21 (DE3) cell line is an ideal industrial host for recombinant receptor expression that exerted several prominent features such as rapid cell growth in minimal medium, low protease abundance and the amenability to high density culture [1]. We transferred the recombinant plasmid of pReceiver-NMDA-2A-EGFR into *Escherichia coli* BL21 (DE3) cells and induced the receptor expression using auto-induction medium and Luria-Bertani (LB) medium containing 0.5 mM or 1.0 mM isopropyl-β-D-1-thiogalactopyranoside (IPTG) with vigorous shaking of 220 rpm under common expression condition of 37 ℃ for 24 h and 36 h. The *Escherichia coli* cells were harvested by centrifugation at 8500 rpm and 4 ℃ for 15 min, and 13.8 g cell pellet was obtained by removing the cell-free culture medium. Moreover, the cell pellet was resuspended in 138 mL phosphate-based buffer pH 7.4 and lysed by intermittent ultrasonication for 40 min at 4 °C with 3 s on and 5 s off. Finally, the cell lysate was centrifugated at 8500 rpm and 4 ℃ for 20 min and the supernatant containing NMDA-2A was collected for receptor immobilization. Sodium dodecyl sulfate polyacrylamide gel electrophoresis (SDS-PAGE) was used to assess whether NMDA-2A had been expressed.

**The methods for SEM and XPS**

The bared microspheres (amino polystyrene microspheres), ibrutinib-modified microspheres and NMDA-2A-modified microspheres were washed gently with water for three times before scanning electron microscope (SEM) and X-ray photoelectron spectroscopy (XPS) analysis. Prior to SEM examination, microspheres were adhered to a platform of carbon film and sprayed with a thin conductive gold film for 5 min. Zeiss Sigma HV SEM (Oberkochen, Germany) with InLens capabilities was used to characterize the surface morphology of microspheres. The accelerating voltage was 5 kV and working distance was 7.2 mm. The micrographs were obtained at 1000-10000× machine magnification. XPS measurements were carried out by Escalab Xi^+^ (Thermo Fisher Scientific, USA). Monochromatic Al Kα radiation (1486.7 eV, 14.8 kV) was used as as incident radiation and the pass energy was 200 eV. The spectral range was from 1350 to 0 eV and quantitative analysis data of different elements (C1s, N1s and O1s) were recorded to evaluate the changes in microsphere surface after ibrutinib or NMDA-2A modification. All XPS Data were analyzed by Thermo Advantage software.

**The method for immunofluorescence studies**

The immunofluorescence studies for bared microspheres, ibrutinib-modified microspheres, NMDA-2A-modified microspheres were conducted in the same condition. Briefly, the microspheres were incubated with rabbit anti-NMDA-2A antibody (Lot: 00083510) diluted 1:400 in TBST at 4 ℃ overnight after washing them with tris-buffered saline Tween-20 (TBST) for three times. Subsequently, the microspheres were washed three times with TBST to remove unbound antibodies and incubated with goat anti-rabbit IgG antibody (CAT: 33116ES60) diluted 1:100 in TBST at room temperature in dark condition for another 2 h. Nikon Eclipse Ts2 (Shanghai, China) was used to visualize the microspheres.

**The specificity and stability characterization of NMDA-2A column**

The void time of NMDA-2A column was determined by sodium nitrite since it had no interaction with NMDA-2A column. Quinidine sulfate, donepezil hydrochloride (the antagonists of NMDA receptor), bisoprolol, and esmolol (the antagonists of β adrenoceptor) were used to assess the specificity of the receptor column. Moreover, we evaluated the stability of the column by checking the peak profile and retention time of quinidine sulfate and donepezil hydrochloride with continuous use over 30 days. The detection wavelength for quinidine sulfate and donepezil hydrochloride was 229 nm, 217 nm for sodium nitrite, 235 nm for bisoprolol and esmolol. The mobile phase was phosphate-based buffer (20 mM, pH 5.7) consisting of 10% isopropanol (V/V) at a flow-rate of 0.4 mL/min.

**The method for frontal analysis**

In this method, the ligand is dissolved in the mobile phase and binds with the affinity stationary phase. Distinct breakthrough curves of diversed ligands are generated due to the difference in their binding abilities to the receptor, which can be utilized to determine the binding affinity between immobilized receptor and ligands. If there was one type of binding sites, the interaction between the receptor and ligands can be described by Eq. (1) [2].

(1)

where *q* is adsorption amount, *m_L_* is the number of binding sites, *K_a_* is the association constant and [*C*] represent the ligand concentration. In the present study, the mobile phase for quinidine sulfate was phosphate-based buffer (20 mM, pH 6.8) consisting of 5% isopropanol (V/V) at concentrations ranging from 15.6 nM to 8 μM (15.6 nM, 31.2 nM, 62.5 nM, 125.0 nM, 250.0 nM, 1.0 μM, 2.0 μM, 4.0 μM and 8.0 μM) with a flow-rate of 0.9 mL/min. The mobile phase for donepezil hydrochloride was from 31.2 nM to 16 μM (31.2 nM, 62.5 nM, 125.0 nM, 250.0 nM, 0.5 μM, 1.0 μM, 2.0 μM, 4.0 μM, 8.0 μM and 16.0 μM) with a flow-rate of 0.2 mL/min.

**The method for injection amount-dependent analysis**

Injection-amount dependent method is another practical methodology for receptor-ligand interaction analysis that developed by our group in 2014 to address the drawbacks of frontal analysis [3]. This model assumes that binding sites are uniformly distributed on stationary phase surface and longitudinal diffusion of chromatographic column can be ignorable. Under these conditions, Eq. (2) can be utilized to investigate the receptor-ligand interaction.

(2)

where *k* is retention factor of the ligand, *n_I_* is the amount of injection solute, *n_t_* is the number of binding sites. In addition, *K_a_* is the association constant and *V_m_* is void volume of chromatographic system. A linear relationship between *kn_L_*/(1+*k*) and *kV_m_* can be observed, and the association constant (*K_a_*) and number of bindings sites can be calculated according to slope and intercept of Eq. (2). In the present study, the mobile phases for quinidine sulfate and donepezil hydrochloride were consistent with those of frontal analysis. In this section, the concentrations for quinidine sulfate were ranging from 10 μM to 320 μM (10 μM, 20 μM, 40 μM, 80 μM, 160 μM and 320 μM) at a flow-rate of 1.0 mL/min. The concentrations for donepezil hydrochloride were were ranging from 20 μM to 640 μM (20 μM, 40 μM, 80 μM, 160 μM, 320 μM and 640 μM) at a flow-rate of 0.4 mL/min. All chromatographic experiments were carried out on the Waters Acquity^TM^ ultra-performance liquid chromatograph (Milford MA, USA).

**Cell culture**

The highly differentiated PC-12 cells (Cat: CL-0481) were purchased from Procell Life Science & Technology Co., Ltd and cultured in serum-free DMEM high glucose medium containing 1% penicillin/streptomycin and 10% fetal bovine serum. The cells were passaged every two days by trypsinization at a split ratio of 1:4 and maintained at 37 ℃ in a 5% CO_2_ atmosphere. After cell adherence, the cells were stimulated with Aβ_25-35_ for 24 h to establish the *in vitro* model of AD and treated with various concentrations of crocetin for another 24 h. The Aβ_25-35_ (Cat. Y-0044) was dissolved in distilled water at a concentration of 25 μM and incubated at 37 ℃ in a 5% CO_2_ atmosphere for four days to form Aβ aggregates before the simulations began.

**Cell viability analysis**

The viability of cells treated with different concentrations of crocetin was determined by 3-(4,5-Dimethylthiazol-2-yl)-2,5-diphenyltetrazolium bromide (MTT) assay. The cells were treated with crocetin at concentrations ranging from 0.6 μM to 80 μM. After 24 h of treatment, 20 μL of MTT solution (5 mg/ml) was added to each well, and the 96-well plate was incubated at 37 ℃ for 4 h. After incubation, dimethyl sulfoxide was added to the wells to dissolve formazan crystals. The 96-well plate were incubated at 37 ℃ for another 15 minutes. After shaking for 5 minutes, the absorbance density values were read using a microplate reader (TECAN, 200 pro, USA) at 570 nm.

**References**

[1] H. Waegeman, S. De Lausnay, J. Beauprez, J. Maertens, M. De Mey, W. Soetaert, Increasing recombinant protein production in Escherichia coli K12 through metabolic engineering, N Biotechnol, 30 (2013) 255-261.

[2] Q. Li, X. Ning, Y. An, B.J. Stanley, Y. Liang, J. Wang, K. Zeng, F. Fei, T. Liu, H. Sun, J. Liu, X. Zhao, X. Zheng, Reliable analysis of the interaction between specific ligands and immobilized β-2-adrenoceptor by adsorption energy distribution, Anal Chem, 90 (2018) 7903-7911.

[3] X. Zhao, Q. Li, C. Xiao, Y. Zhang, L. Bian, J. Zheng, X. Zheng, Z. Li, Y. Zhang, T. Fan, Oriented immobilisation of histidine-tagged protein and its application in exploring interactions between ligands and proteins, Anal Bioanal Chem, 406 (2014) 2975-2985.


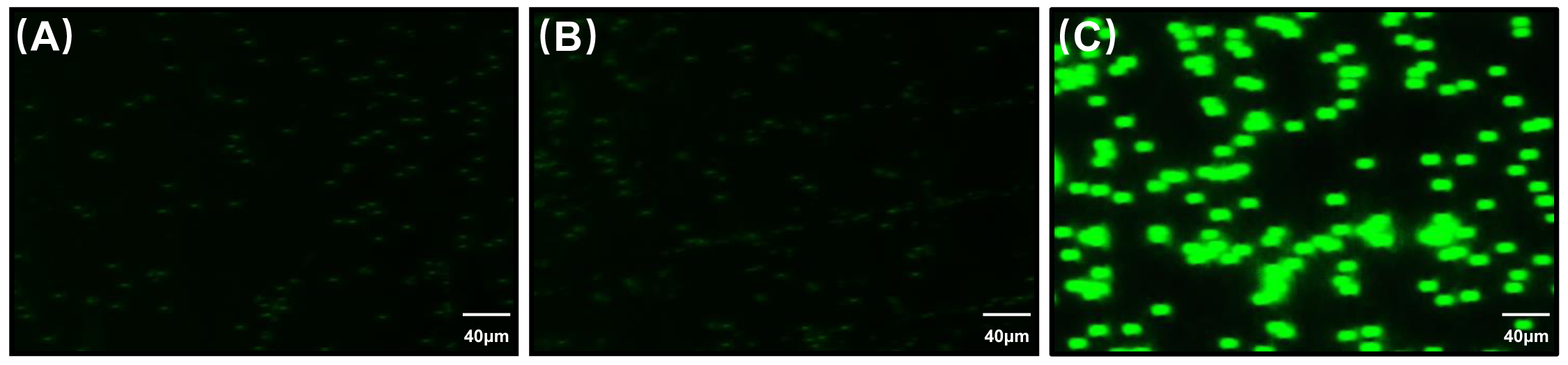
Fig. S1 The immunofluorescence studies of bared microspheres (A), ibrutinib-modified microspheres (B), NMDA-2A-modified microspheres (C).


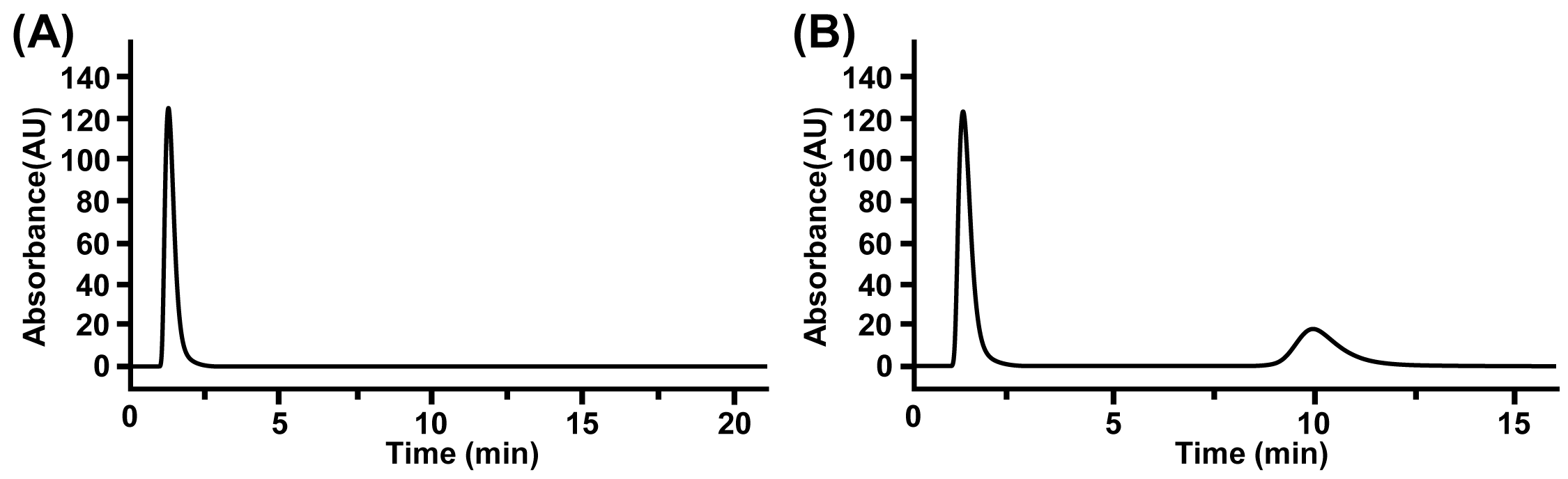


Fig. S2 The reliability characterization of NMDA-2A column by a mixed sample. The chromatographic peak of T0070907, bisoprolol, crocin I, and ambrisentan (the non-binding ligands for NMDA receptor) on NMDA-2A column (A); the chromatographic peak of T0070907, bisoprolol, crocin I, ambrisentan (the non-binding ligands for NMDA receptor), and huperzine A (a ligand of NMDA) on NMDA-2A column (B).


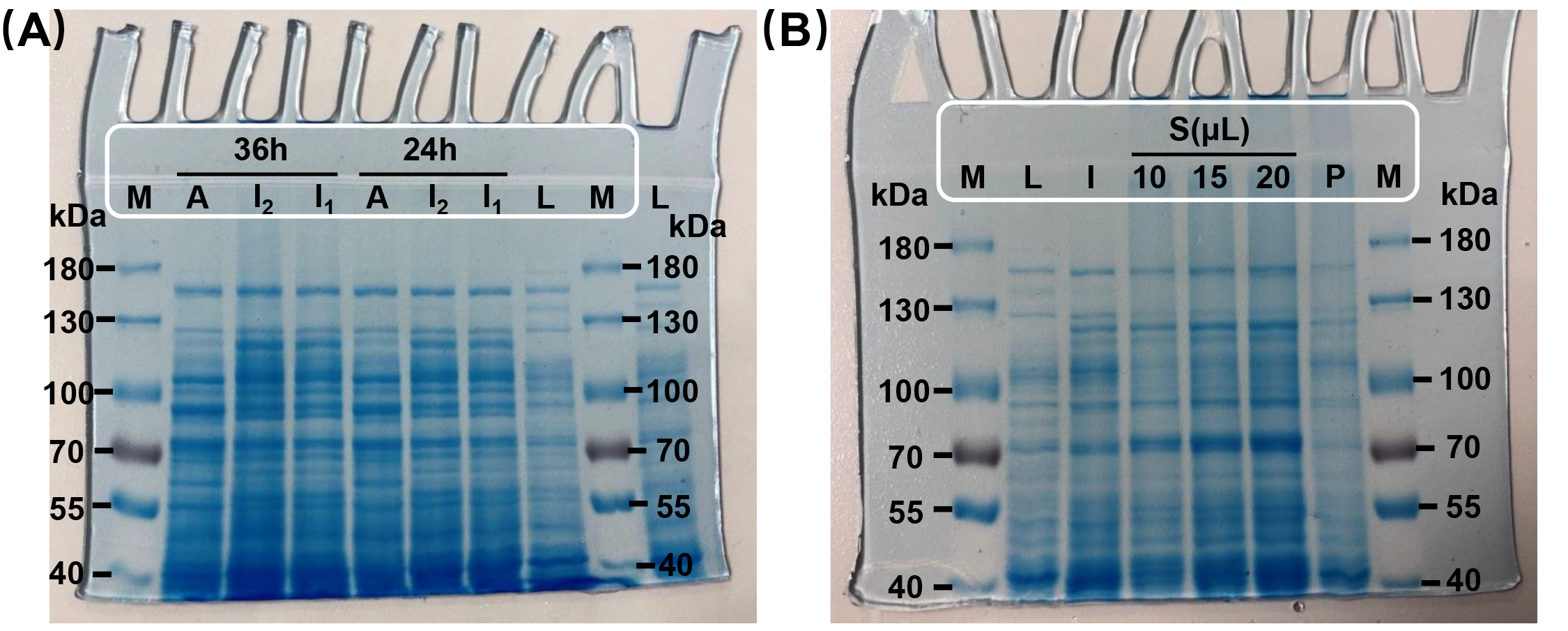


Full unedited gel for Fig.1. Lanes of the unedited gels that appear in Fig.1 were highlighted in white squares.


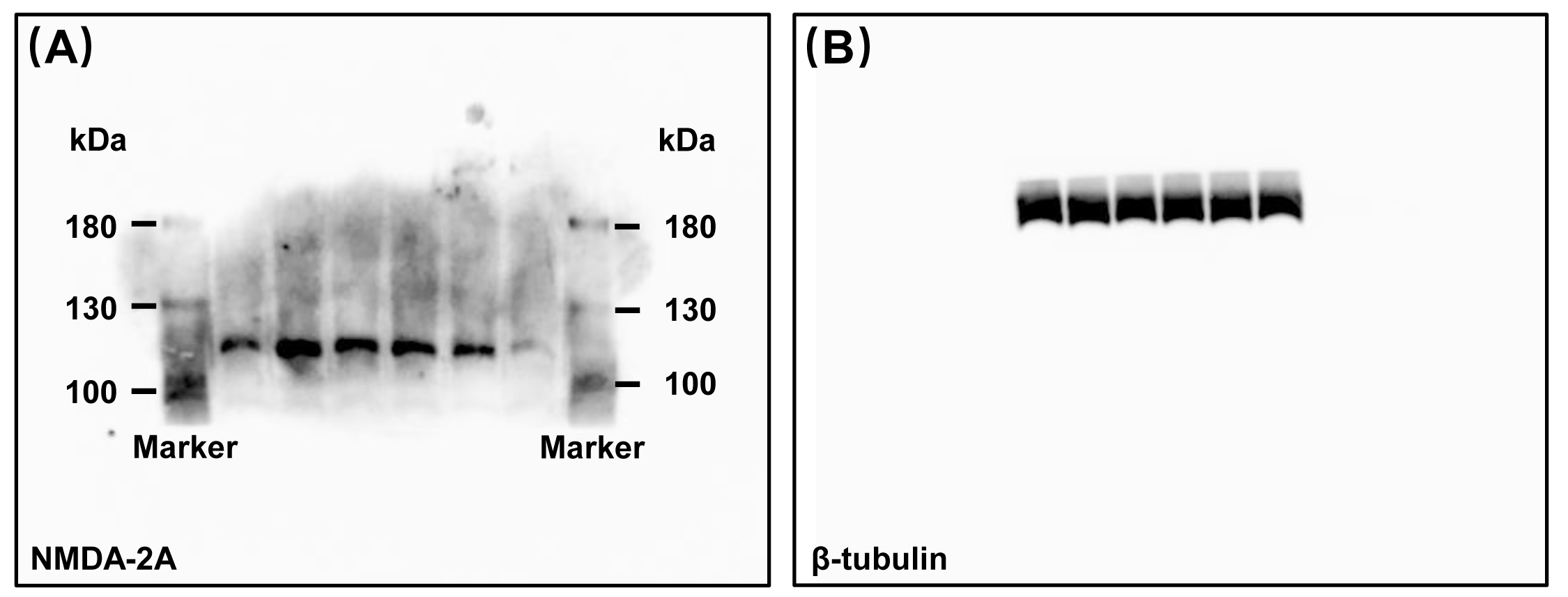


Full unedited blot for Fig. 6I. Except for lanes for markers, all lanes of the unedited blots were used in Fig.6I.
